# Supplementary material for: Oxidative Stress-Induced Pentraxin 3 Expression Human Retinal Pigment Epithelial Cells Is Involved in the Pathogenesis of Age-Related Macular Degeneration
Source: Int J Mol Sci. 2019 Nov 29;20(23):6028. doi: 10.3390/ijms20236028 (PMC6928709; doi:10.3390/ijms20236028)
Supplement: Supplementary file 1 [file ijms-20-06028-s001.pdf]

International Journal of
Molecular Sciences

MDPI

Supplementary Results

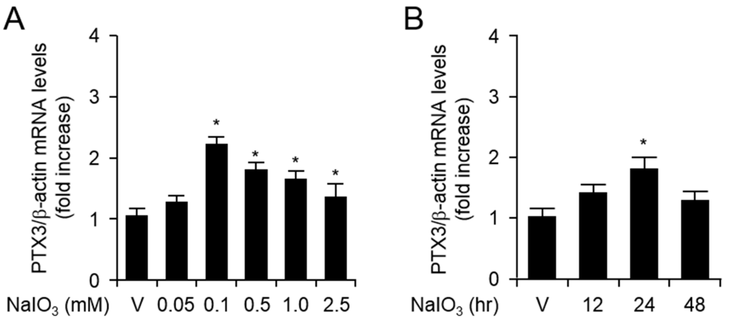

Figure S1. The expression of pentraxin 3 (PTX3) mRNA levels was enhanced after $NaIO_3$ administration in human RPE cells. Human retinal pigmented epithelial cell lines ARPE-19 cells were treated for 24 h in various doses of $NaIO_3$ (**A**). ARPE-19 cells were exposed to 100 μM $NaIO_3$, for the indicated time points (**B**). Values are presented as mean ± SD, n=3. **p* < 0.05, increased PTX3 mRNA expression after $NaIO_3$ administration vs. vehicle (V).

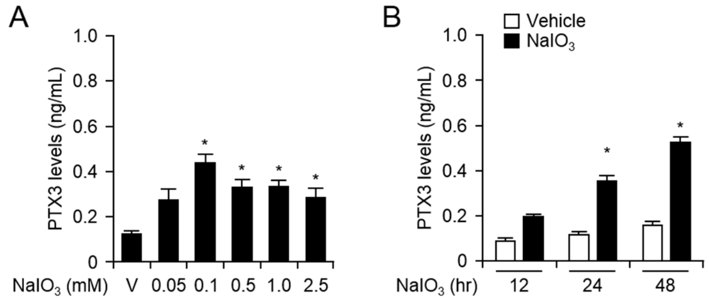

Figure S2. The protein levels of PTX3 were enhanced after $NaIO_3$ administration in human RPE cells. Human retinal pigmented epithelial cell lines ARPE-19 cells were treated for 48 hours in various doses of $NaIO_3$ (**A**). ARPE-19 cells were exposed to 100 μM, for the indicated time points supernatants were harvested and analyzed for PTX3 production (**B**). Values are presented as mean ± SD, *n* = 12. **p* < 0.05, increased PTX3 after $NaIO_3$ administration vs. vehicle (V).

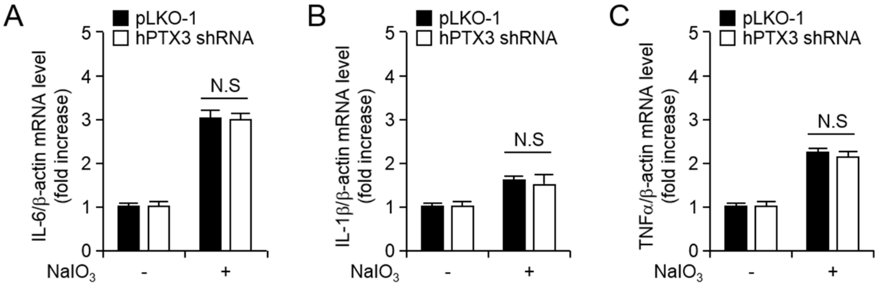

Figure S3. The mRNA levels of inflammatory cytokines were not enhanced hPTX3 shRNA expressing ARPE-19 cells in response to $NaIO_3$. Total RNA was extracted from control or hPTX3 shRNA expressing ARPE-19 cells 12 h after 100 μM NaIO3 administration. mRNA levels of IL-6 (**A**), IL-1β (**B**) and TNFα (**C**) were analyzed by quantitative real-time RT-PCR. Human β-actin was used as a control for normalization. Expression levels of each mRNA are divided by expression of β-actin and shown as a ratio of each mRNA/β-actin. Values are presented as mean ± SD, n = 3. N.S. indicates non-significance.
